# The RESET Mindset Model applied on decreasing antibiotic usage in dairy cattle in the Netherlands

**DOI:** 10.1186/s13620-017-0085-x

**Published:** 2017-02-23

**Authors:** T. J. G. M. Lam, J. Jansen, R. J. Wessels

**Affiliations:** 1 Communication in Practice, Nijmegen, The Netherlands; 2 GD Animal Health, Deventer, The Netherlands; 3 Department of Farm Animal Health, Faculty of Veterinary Medicine, Utrecht University, Utrecht, The Netherlands; 4 St Anna Advies, Nijmegen, The Netherlands

**Keywords:** RESET, Mindset, Antibiotics, Dairy

## Abstract

**Background:**

Prudent use of antibiotics is important to prevent antibiotic resistance in humans and in animals. For this reason politicians demanded a decrease of total antibiotic use and of use of critically important antibiotics in animal husbandry in the Netherlands. In the dairy sector the use of antibiotics almost halved in the years 2009–2015, with a decrease of the use of critically important antibiotics to very low levels.

**Theory of behaviour change:**

To realize a sustainable decrease in antibiotic usage, the mindset towards the subject was considered crucial. Based on several models from social psychology, the RESET Mindset Model was used. This model contains the most important cues to change human behaviour, being Rules and regulations, Education and information, Social pressure, Economics, and Tools. To change behaviour of groups in order to reach a tipping point, it is of utmost importance to not choose among the different cues, but to use them all.

**Changing antibiotic usage in dairy cattle:**

In order to decrease antibiotic usage in dairy cattle in the Netherlands several actions, obliged as well as voluntary, were undertaken. An independent veterinary medicine authority was founded that became active for all animal sectors. In the dairy sector a national database on antibiotic usage called MediRund was developed, which made transparency and benchmarking on antibiotic usage at the national and the herd level possible. Several other activities are described, such as herd health and treatment plans, selective dry cow therapy, and the strong limitation on the use of critically important antibiotics. Antibiotic usage at the herd level, referred to as the ‘antibiotic number’, became an important and socially accepted herd level parameter.

**RESET the dairy farmer’s mindset on antibiotic usage:**

The actions undertaken worked through different cues, all part of the RESET Mindset Model. As such, different types of dairy farmers sensitive to different types of cues were motivated to change their behaviour.

**Conclusion:**

Antibiotic usage in dairy cattle in the Netherlands decreased significantly by intense cooperation between the most important stakeholders in the dairy chain, taking communication seriously and applying the RESET Mindset Model.

## Background

Since the second world war, antibiotics became widely available to cure bacterial diseases, which had an enormous impact on lifetime expectancy of people suffering from bacterial infections. Antibiotics were also used in animals, initially to cure diseases, later also to prevent diseases and as growth promoters [1], which led to an enormous increase in antibiotic usage in the livestock industry in, among other countries, the Netherlands [2]. The availability of antibiotics was also of great importance for the improvement of animal health in the dairy industry, specifically related to udder health [3] and to a less extent in claw health [4] and uterine diseases [5]. Due to the simple fact that most milk is used for products such as cheese and yoghurt, and antibiotic residues have a negative effect on that production process, antibiotic use in lactating cows has always been limited to situations in which it was considered unavoidable.

In 2008 antibiotic usage in animal husbandry became a political issue in the Netherlands. Whereas antibiotic usage in humans was relatively low compared to other European countries, antibiotic usage in the Dutch livestock industry was relatively high [6, 7]. After a number of incidents with methicillin resistant *Staphylococcus aureus* (MRSA) and extended spectrum beta-lactamase producing bacteria (ESBLs) in animals [8, 9] antibiotic resistance became an important issue on the political agenda. With the objective to reduce antibiotic resistance, reduction goals for antibiotic use in animals were set by the Dutch government. The goal set was a decrease of 20% in 2011, 50% in 2013, followed by 70% in 2015, all as compared to 2009 [10]. At the same time it was indicated that the livestock industry itself had the responsibility to realize this reduction.

At that time it was clear that, although the dairy sector was not the sector in which most antibiotics were used or that seemed to have a big antibiotic resistance problem, there were some issues there too that had to be solved [11]. In December 2008 a taskforce on antibiotic use in cattle (TAUC) was established, as was done in other species. In the TAUC all major stakeholders were represented, being representatives of the farmers organisations, the dairy and meat plants, the veterinarians as well as some technical experts. The challenge for the TAUC was to realize a reduction in antibiotic use although usage was relatively low already [11] and antibiotic resistance was not perceived as a problem by many farmers and veterinarians [12]. Additional challenges were to change practices such as blanket dry cow treatment (DCT) and extended treatment of (sub)clinical mastitis, which had been promoted over the years, as was the use of zero-withdrawal products, which were widely available and used. Finally, the TAUC did not want the change in antibiotic policy to have a negative impact on animal health and welfare.

**Fig. 1:**
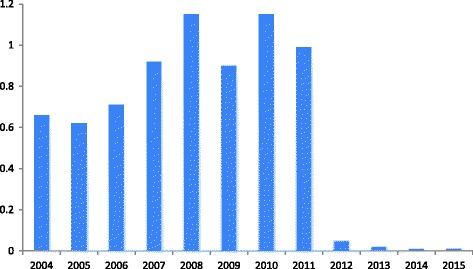
Use of critically important antibiotics in dairy cattle in the Netherlands in 2004–2015, expressed in defined daily dosages of antimicrobials (DDDA_NAT_) [2, 12]

The use of antibiotics in the dairy sector decreased by 47% in the period 2009–2015, with a decrease in the use of critically important antibiotics to very low levels [13], as presented in Fig. 1. In 2014, antibiotic DCT was used in 61% of cows dried off [14], where it was 94% in 2009 [15], without deteriorating udder health [14]. The mindset of dairy farmers with respect to the reduction of antibiotic usage in general and in the dairy sector was generally positive [16].

In this review paper we use the strategy of the TAUC as an example to show how the RESET Mindset Model can be used to change behaviour of dairy farmers. The specific issues involved in the communication process and the decisions that were made were described, as was the approach to change the mindset of dairy farmers and veterinarians towards reduction of antibiotic usage in dairy cattle in the Netherlands.

### Theory of behaviour change

An important step in the success model of the reduction of veterinary antibiotic use in the Netherlands [10] was that recognition of the fact that - to change behaviour related towards antibiotics - not only the knowledge of dairy farmers and veterinarians had to improve, but also their mindset towards the subject. To actually improve behaviour, two requirements have to be fulfilled. The first is that one knows what to do, what the optimal behaviour is, and the second is that one is motivated enough to implement that behaviour [17]. In veterinary medicine we tend to focus on the former part, the technical content [18]. Traditionally veterinarians assume that dairy farming is an activity executed primarily based on rational, technical, and economic considerations [19]. Although well-balanced choices are crucial in farm management, we learned in mastitis studies that management on dairy farms is hardly ever fully rational [20]. When trying to understand farmer motivation and behaviour, much can be learned from social psychology. One of the most used theories to understand people’s behaviour is the Theory of Planned Behaviour, described by Ajzen [21] and by Fishbein and Yzer [22]. In short, this model says that if someone is actually willing to solve an issue, if he is positively influenced by important peers and if he has the feeling he can control and perform his actions, he will have a positive intention and probably will change his behaviour. Another theory about drivers for implementing preventive disease measures is the Health Belief Model of Janz and Becker [23]. This model has for instance been described to fit with behaviour related to preventive management on dairy farms [24] and in human health [25]. Farmers were found to be motivated to change their mastitis management if they perceived a serious mastitis problem and if they saw effective solutions. Solutions were only considered effective if they were easy applicable and if the benefits were expected to outweigh the barriers to perform the proposed management measures [18]. These models assume that farmers make rational decisions on their daily activities. Many farmers, however, do not approach their decisions on daily routines that rationally [24]. Therefore, peripheral strategies to change behaviour, such as using tools, cues and nudges, may be useful to unconsciously steer people towards the desired behaviour [22]. This approach has also been proven effective for dairy farmers [20].

**Fig. 2:**
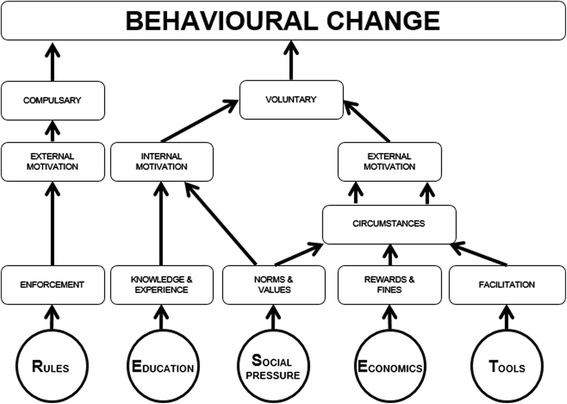
The RESET Mindset Model, adapted from Woerkum et al. [21]

Based on the Theory of Planned Behaviour [21, 22], the Health Belief Model [23], the Elaboration Likelihood Model of Persuasion [26] and earlier work of van Woerkum et al. [27] and Leeuwis [28], we developed a model that is easy to use in practice, the RESET Mindset Model [29] as is presented in Fig. 2. This model was advised to the TAUC to use as framework to develop strategies to decrease antibiotic usage in the Netherlands. The RESET Mindset Model summarizes different models from literature in five important cues to action: Rules and regulations, Education and information, Social pressure, Economics, and Tools. People can be motivated to change their behaviour by one or more of these cues. Although veterinarians often think it hard to change behaviour of their clients, studies showed that using different communication strategies may help [18, 26] and decreases the number of hard-to-reach-farmers [30]. Taking the farmers preferred learning style into account as well as the cues of action that he or she may be sensitive to, certainly will help in effective communication to change behaviour [24]. Consequently, to reach as much people as possible in national animal health programs, one shouldn’t chose among the different cues, but use them all simultaneously. With such a differentiated approach, using of a mix of stimuli, one will reach different types of people while at the same time increasing motivation, trying to get group behaviour over a tipping point.

It is clear that the different cues of the RESET Mindset Model are interrelated, and that people are often motivated by the various cues simultaneously. Nevertheless, for practical reasons, different cues will be discussed separately below.

#### R for rules and regulations

Generally rules are intended to force you to perform a desired behaviour. Compulsory behavioural change is enforced by regulations and restrictive provisions [27]. Thus, if rules are clear and if there is a system for monitoring and enforcement in place, they can be very effective [31]. Compulsory behavioural change, when used as the only approach will, however, only last as long as the coercion or law enforcement exists. Therefore it is preferably accompanied by voluntary behavioural change, which is based on either internal or external motivation.

#### E for education and information

Education is a very strong method to increase internal motivation. Internal motivation is not easy to influence, but once it is done, it still is not easy to change, which we then consider a positive effect. Veterinarians generally tend to overestimate the effect of the educational approach, based on the assumption that once the technical arguments are given, farmers take rational decisions and will act accordingly [18, 19]. The idea is that once a farmer will understand ‘how and why’, behaviour will change. This certainly is true for some farmers, but if it is the only approach, it will definitely not be sufficient to motivate the majority [26].

#### S for social pressure

Probably the most important factor in changing behaviour is social pressure. Most people would do anything in their power to become and remain part of a certain group. The urge to belong is an important motivator for behavioural change [32]. Veterinarians and other advisors play an important role in shaping this societal frame of reference, because they have a strong influence on farmers’ opinions about animal health [18]. People mimic behaviour from groups and role models [33], although there may be no scientific evidence that supports that behaviour. A nice example from the dairy industry is postmilking teat disinfection. Although the working mechanism of teat disinfectants are the same all over the world, the way they are applied on cows teats differs. In order to be effective the disinfectant should cover the teats. The method of achieving this may, however, differ. In the United States dipping is considered the best method, while that is spraying in New Zealand. In review reports on teat disinfection in the United States spraying is not even mentioned as an option [34], whereas in New Zealand it is the other way around, and dipping is outside of the scope [35]. Why? Because that is the way people do it, it’s the unwritten social norm.

#### E for economics

One of the ways to externally motivate people is through changing circumstances that have economic consequences. Many advisors think money is the only factor influencing farmers’ decisions. Although this is seldom true, economic consequences undoubtedly are important in a free-market economy. That may be either way, positive as well as negative, profits and costs, bonuses and penalties, which have been mainly described for bulk milk somatic cell count (BMSCC) [36]. Economic effects seem to be most powerful if the effect can be sensed directly. Farmers were found not to be too impressed by calculations of lost profit through missed production [37] whereas they were sensitive to penalties, and to a lesser extent for bonuses on BMSCC [36].

#### T for tools

One of the important factors influencing circumstances are technical facilities that make it easier to perform the desired behaviour. These tools can also be design solutions or nudges, that unconsciously steer people in the right direction, without them being aware [38]. Using tools as a mean of external motivation is often part of a peripheral communication strategy [20]. Tools can be very important to take away barriers to change behaviour as described in the Health Belief Model [23]. Milking with clean hands became much easier through the use of milking gloves [20] which, intended or not, had a beneficial effect on bacterial counts on hands [39].

### Changing antibiotic usage in dairy cattle

The main goal of the TAUC was to improve the antibiotic resistance situation through decreasing the amount of antibiotics administered while increasing prudent antibiotic usage. There were some specific conditions that had to be fulfilled such as limiting the use of critically important antibiotics and banning the preventive use of antibiotics as in applying blanket DCT. To realize these goals, several activities were initiated, of which two were obligatory for all farmers: transparency of antibiotic use at the herd level, and implementation of a herd health and treatment plan [10]. These obligatory activities were also implemented in the dairy sector, were accompanied by a number of other activities and supported by a communication plan to motivate farmers and their veterinary practitioners to optimise cattle health and decrease antibiotic usage as much as possible. In 2010 an independent Veterinary Medicine Authority (SDa) was founded, consisting of a panel of experts with a background in microbiology and epidemiology, both human and veterinarian. Their task was to monitor national trends in antibiotic use in animal husbandry based on data delivered by the different livestock sectors, and to judge if their obligations were fulfilled [10].

To make antibiotic usage transparent at the herd level calculation of the Defined Daily Dose of Antimicrobials (DDDA) was introduced basically indicating the number of days of antimicrobial treatment per animal per year. The DDDA was calculated at the national (DDDA_NAT_) as well as at the farm level (DDDA_F_), and was specified for specific types of antibiotics, age groups and application methods, as described earlier by Santman-Berends et al. [40]. The data that were used for this purpose were based on the amount and type of antibiotics delivered by a veterinary practitioner to a specific farmer, based on established 1-to-1 relationships [10]. These data were collected in a national database called MediRund, that was developed by the TAUC. The SDa used the MediRund data for their monitoring activities at the national level, and to set signaling and action thresholds based on which high use herds and veterinarians can be identified by the involved quality systems [2]. Farmers receive every three months an overview from MediRund, containing their specific farm situation, including a benchmark based on the national average and signaling and action thresholds using colour codes. Red means the action threshold is passed, and immediate action is needed. The dairy farmer and his veterinarian are obliged to develop a plan to solve the problems, otherwise the milk will no longer be collected by the dairy plant. Orange means the signalling threshold is passed and indicates there is room for improvement. Green finally means that antimicrobial use is at an acceptable level and no further action is required at that point.

The herd health plan is based on the idea that if diseases are better controlled the need for antibiotics reduces. Every dairy farmer is obliged to choose one certified cattle veterinarian (1-to-1 relationship) [10], with whom he shares responsibility on prudent antibiotic usage on the dairy farm. In cooperation with the farmer the veterinarian makes a herd health plan that they both agree on to execute. The content and implementation of that plan should be evaluated at least on an annual basis. The herd health plan consists of a herd level treatment protocol and a preventive part. The treatment protocol is intended to optimize antibiotic treatments executed by the farmer himself and contains recommendations on antibiotic use for major indications on the dairy farm such as clinical mastitis and lameness. These recommendations are based on the available information on bacteria and sensitivity patterns from the herd, and on the national guidelines of the Working Group on Veterinary Antibiotic Policy (WVAB) of the Royal Dutch Veterinary Association. In the preventive part of the herd health plan animal health and antibiotic usage in the previous year is monitored. Additionally the management situation of the herd is evaluated based on infection pressure as well as host resistance, covering issues such as biosecurity, pathogen transmission, feeding, housing and milking.

Most of the antibiotics used in dairy cattle are administered via the intramammary route. In the Netherlands this accounted for approximately 70% of total antibiotic usage of which roughly 1/3 in mastitis treatment and 2/3 in DCT [41]. Because of withdrawal-time of antibiotics used to treat mastitis in lactating cows, there is an intrinsic limitation to the use of this type of antibiotics. For obvious reasons this is not the case in DCT. Based on the principle that preventive use of antibiotics was no longer allowed, from 2013 onward blanket DCT was forbidden, where it had been fiercely promoted in earlier years [16]. Based on cow-level studies done in Dutch circumstances, it was expected that selective DCT would lead to an increase of clinical and subclinical mastitis [42]. In 2014 a guideline was launched by the Royal Dutch Veterinary Association, which stated that dry cow antibiotics were only allowed after intramammary infections (IMI) were diagnosed at drying off. As indication of IMI, somatic cell count (SCC) can be used. It was found that the cut-off levels of SCC did influence the effect of selective DCT on the incidence of clinical and subclinical mastitis at the cow-level [40], while the effect of the exact value of these cut-off levels was limited at the herd-level [43].

Apart from the quantitative approach of total antimicrobials used the types of antibiotics received specific attention. The relation between antibiotic usage and antibiotic resistance has been shown in general [44] as well as for specific antibiotics in dairy cattle. In the United Kingdom and in the Netherlands it was found that herds that used third or fourth generation cephalosporins were almost four times more likely to have ESBL producing *E. coli*, while no association was found with other antimicrobials [45, 46]. Antibiotics used in animal husbandry were subdivided in three categories, with increasing likeliness to give rise to development of antibiotic resistance: veterinary important, veterinary highly important, and veterinary critically important antibiotics [47]. Veterinary important antibiotics were defined as antibiotics that are considered to be effective for the specific indication and don’t induce resistance by ESBL/AmpC production [48]. If infections need to be treated with antibiotics, this type of antibiotics ought to be used. If, however, no suitable antibiotic of this type is available, veterinary highly important antibiotics may be used. To be allowed to use highly important antibiotics, there should be a specific documented reason like patient-history, sensitivity pattern or clinical urgency. Critically important antibiotics finally, are those antibiotics that are of importance for treating multiresistant bacteria in human patients, such as third and fourth generation cephalosporins, some fluoroquinolones and modern long acting macrolides. The use of critically important antibiotics is only allowed for individual animals, when bacteriological culture and sensitivity patterns showed there is no alternative [48].

A final important aspect of antibiotic use in the dairy industry is residue handling. Liquid milk and milk that is processed are monitored very well, leading to a very low percentage of positive findings. In the past, however, very little attention was given to residues in waste-milk. This milk, that contained antibiotic residues in approximately 70% of samples [49] was often fed to young calves despite the potential induction of antibiotic resistance [50]. Feeding waste-milk is not allowed, is not wise and although hard to check, has been highly discouraged.

### RESET the dairy farmer’s mindset on antibiotic usage

To reduce the use of antibiotics in dairy cattle, the RESET Mindset Model was applied, trying to use as many cues as possible to motivate dairy farmers as well as veterinarians to change their behaviour towards antibiotic use. These activities are summarized in Table 1. We do realize that many activities work through different cues, but for reasons of clarity, we decided to choose the main effects as estimated by us.

**Table 1:** The most important simultaneous RESET actions taken by involved stakeholders to decrease antibiotic usage in dairy cattle in the Netherlands

Rules	Education	Social Pressure	Economics	Tools
- 1-to-1 relationship dairy farmer and veterinarian - No preventive antibiotic usage (no blanket DCT) - Herd health plan - Transparency on antibiotic usage - Limitations on use of specific antibiotics - Action plan when antibiotic usage is too high	- Publications in scientific and farmer journals - Press releases - Guidelines on antibiotic usage - Specific courses for veterinarians on herd health plans - Study groups on antibiotic usage for farmers - Lectures, meetings, symposia	- Public opinion on responsibility towards human health - Initiation of the ‘antibiotic number’ DDDA_F_ - Benchmark on DDDA for farmers and veterinarians - Discussions on alternative (preventive) approaches with different herd health advisors	- Costs of dry cow antibiotics - Imminent threat of sanctions when failing to commit - Indirect threat of losing customer trust, national and international	- Herd health and treatment plan - Medi-Rund - Standard treatment protocols - Colour codes for passing signalling and action thresholds on antibiotic usage - Setting signalling and actions thresholds on antibiotic usage

The R was first of all fulfilled by the quantitative reduction goal set by the government. This goal, however, was intangible for the individual dairy farmer and veterinarian. The earlier described 1-to-1 relationship between farmer and veterinarian [10], the obligatory herd health and treatment plan, the ban on preventive use of DCT and on the use of critically important antibiotics had a more direct effect on the individual farm. The transparency of usage of antibiotics (DDDA_F_) with the accompanying signaling and action thresholds, and obligatory action plans when needed also had their direct effect on farm. The guidelines on the use of antibiotics at drying off, and the herd health and treatment plans were, although obligatory, also examples of structured management approaches that were considered helpful by some farmers and veterinarians [16].

The E of education and information was used in many studies done under Dutch circumstances and the scientific publications of these studies [14, 16, 40, 42, 43]. The results of these studies were, simultaneous with other information on this subject, used by the various stakeholders in i.e. information meetings, study groups, lectures and farmers journals. Interestingly enough, although veterinarians should be well aware of the importance of preventing antibiotic resistance, it was in the past hardly ever discussed with farmers [12]. Many farmers indicated they never heard about the potential effect of certain types of antibiotics on antibiotic resistance in animals and humans, including themselves. Parameters that were considered important with respect to the usage of antibiotics were treatment efficacy, withdrawal time and costs. Veterinarians nor other advisors talked about the effect of suboptimal use of antibiotics on antibiotic resistance before.

One of the ways the S is reflected is in a changed social environment of farmers. In most western countries society has changed in a few generations form a mainly agricultural society to one in which farmers are a minority. That has had its consequences on the way the general public thinks of farming and also on the way farmers think of farming. Those changes occur very slowly and differ from region to region but they do occur. The emotional impact of farmers standing up at meetings to tell about their experiences in hospital where they were isolated for suspicion on MRSA because they came from a farm is an indication of that. The transparency of the total antibiotic usage in dairy farms may also have had an effect on social pressure. Veterinarians and other advisors on herd health, such as representatives from the food industry, considered antibiotic use an important parameter and talked about alternative approaches for disease prevention. The DDDA_F_ or the ‘antibiotic number’ as farmers call it became one of the parameters dairy farmers know and that many of them consider an important characteristic.

The effect of social pressure is visible once farmers proudly tell in public meetings they have a low antibiotic number and are among the best in the benchmark. The obliged herd health and treatment plan forces farmer and veterinarian to work together and works also as social pressure for both of them [12]. Other herd health advisors, for example advisors on feed, also are important players in the social network. They had influence because they saw a market opportunity for feed additives that were assumed to have positive effects on dairy health, such as vitamins, minerals, probiotics and certain oils.

The E of economics started having its effect after the initial fear for the effect of using less antibiotics, specifically in DCT, had disappeared. At the herd level using less antibiotics at drying off had minimal effects of the incidence of mastitis but did have an effect on the use of dry cow antibiotics [43] and the associated expenditures. Farmers indicated this was one of the aspects they weighted in their decisions on DCT [16] as has been described before [51]. Another indirect economic driver was the imminent threat for political sanctioning if the dairy chain failed to commit to the goals set. Veterinarians were pressured by the possibility to lose their pharmacy as source of income and farmers were insecure about possible future sanctioning.

The T of tools was applied through the above discussed herd health and treatment plans, the guidelines of antibiotic use of the WVAB and on antibiotic use in selective DCT, and the MediRund data that were visualized in their DDDA_F_ using the colour codes described. These tools had their effect through other cues, but also directly as technical devices facilitating the desired behavioural change.

All the described activities together (Table 1) combined different cues to change the mindset of dairy farmers and veterinarians towards antibiotic usage on dairy farms. As described by Jones et al. [51], cooperation of the most important stakeholders on dairy farms seems crucial in sending the same message to dairy farmers and underlines the importance of the subject. Although a control group is lacking, we are convinced that this integrated approach played a crucial role in the enormous decrease of antibiotic usage in dairy cattle in the Netherlands. Despite the fact that information provided on DCT and on critically important antibiotics was contradicting to earlier provided information by veterinary practitioners and in national projects [15], most farmers were convinced that SDCT and the selective use of antibiotics in general were a sound approach [16]. This shows that using the RESET Mindset Model which combines multiple communication strategies, can change ingrained behaviour patterns. The behaviour of dairy farmers in the Netherlands towards antibiotic usage has changed, which seems to be based on an actual change of mindset and therefore likely will be successful on the long term.

## Conclusion

Antibiotic usage in dairy cattle in the Netherlands decreased significantly by intense cooperation between the most important stakeholders in the dairy industry, taking communication seriously and applying the RESET Mindset Model.

## Abbreviations

DCT: Dry Cow Treatment
DDDA: Defined Daily Dose of Antimicrobials
IMI: Intramammary Infection
RESET: Rules and regulations, Education and information, Social pressure, Economics, Tools
SCC: Somatic Cell Count
SDa: Veterinary medicine authority (Stichting Diergeneesmiddelen autoriteit)
TAUC: Taskforce on Antibiotic Use in Cattle
WVAB: Working Group on Veterinary Antibiotic Policy
